# Association between triglyceride glucose index-related indices and kidney stones in adults based on NHANES 2007–2020

**DOI:** 10.3389/fendo.2024.1516982

**Published:** 2025-01-07

**Authors:** Ming Liu, Ping Yang, Yunpeng Gou

**Affiliations:** Department of Pediatric Surgery, Suining Central Hospital, Suining, Sichuan, China

**Keywords:** kidney stones, TyG index, TyG-BMI, TyG-WC, TyG-WHtR, insulin resistance, NHANES

## Abstract

**Background:**

The triglyceride-glucose (TyG) index and related indices, including the triglyceride-glucose body mass index (TyG-BMI), triglyceride-glucose waist circumference (TyG-WC), and triglyceride-glucose waist-to-height ratio (TyG-WHtR), are increasingly recognized as valuable markers of insulin resistance (IR). This study aimed to assess the associations between these TyG-related indices and kidney stones.

**Methods:**

This cross-sectional study analyzed data from 10,824 participants obtained from the National Health and Nutrition Examination Survey (NHANES) conducted between 2007 and 2020. Weighted logistic regression models were employed to evaluate the associations between TyG-related indices and kidney stones, with adjustments for potential confounding factors. Subgroup analyses and smooth curve fittings were performed to further examine these associations, while receiver operating characteristic (ROC) curves were used to compare the predictive performance of each index.

**Results:**

All TyG-related indices demonstrated significant positive associations with kidney stones when analyzed as continuous variables. The odds ratios (OR) with 95% confidence intervals (CI) were 1.0040 (1.0028, 1.0052) for TyG-BMI, 1.0015 (1.0011, 1.0020) for TyG-WC, and 1.3305 (1.2277, 1.4419) for TyG-WHtR. Similar trends were observed in subgroup and smooth curve analyses. When stratified into tertiles, higher tertiles of each TyG-related index were associated with increased odds of kidney stones. TyG-WC demonstrated the strongest predictive capability for kidney stones (AUC = 0.6158), followed closely by TyG-WHtR (AUC = 0.6156) and TyG-BMI (AUC = 0.5949), with TyG showing the lowest AUC (0.5815).

**Conclusion:**

This study identified significant positive associations between TyG-related indices and kidney stone formation. Among these indices, TyG-WHtR exhibited the highest predictive power for identifying kidney stone risk.

## 1 Introduction

Kidney stones are a prevalent urological condition with a substantial global impact. The incidence of kidney stones has been steadily rising worldwide, with estimates ranging from 5% to 19% across various regions (1, 2). In the United States, the prevalence has nearly tripled over recent decades, increasing from 3.2% to 8.8%, particularly in developed nations owing to changes in diet and lifestyle (3). The economic burden is substantial, with healthcare costs related to nephrolithiasis exceeding $10 billion annually (3, 4). Recurrence rates are high, with nearly 50% of individuals experiencing a repeat episode within five years (5, 6). Kidney stones are associated with complications such as infections, renal colic, and chronic kidney disease (7, 8), highlighting the need for effective preventive strategies.

The triglyceride-glucose (TyG) index is a recognized marker of insulin resistance (IR), calculated using fasting triglyceride and glucose levels (9). Recently, new indices combining TyG with anthropometric measures, such as triglyceride-glucose body mass index (TyG-BMI), triglyceride-glucose waist circumference (TyG-WC), and triglyceride-glucose waist-to-height ratio (TyG-WHtR), have been proposed to provide a more comprehensive assessment of metabolic risk by integrating both metabolic and body composition data (10). These TyG-related indices have demonstrated stronger correlations with IR than the TyG index alone (11–13). Since IR has been linked to kidney stone formation (14), TyG-related indices may also be associated with nephrolithiasis. However, despite the increasing evidence on the relationship between IR and kidney stones, only a limited number of studies (15, 16) have examined the association between TyG-related indices and the risk of kidney stones, leaving this area of research relatively underexplored.

This study aims to investigate the association between TyG-related indices and the risk of kidney stone formation. By analyzing data from the National Health and Nutrition Examination Survey (NHANES), we aim to determine whether higher TyG-related indices are predictive of kidney stone risk in adults. This research addresses a gap in understanding the relationship between IR markers, such as TyG-related indices, and kidney stone development, with potential implications for early detection and prevention strategies.

## 2 Materials and methods

### 2.1 Data source

This cross-sectional study analyzed data from the NHANES, conducted between 2007 and 2020. Administered by the Centers for Disease Control and Prevention (CDC) and the National Center for Health Statistics (NCHS), NHANES aims to assess the health and nutritional status of the non-institutionalized U.S. population using a stratified, multistage probability sampling technique. Data collection involved in-home interviews conducted by trained professionals and comprehensive health examinations, including laboratory tests performed at mobile examination centers. The NHANES protocol was approved by the NCHS Ethics Review Board, and all participants or their guardians provided written informed consent. This study adhered to the principles of the Declaration of Helsinki. No additional Institutional Review Board approval was required for this secondary analysis. Further details regarding NHANES methodology are available on the NHANES website (https://www.cdc.gov/nchs/nhanes).

### 2.2 Study population

**Figure 1:**
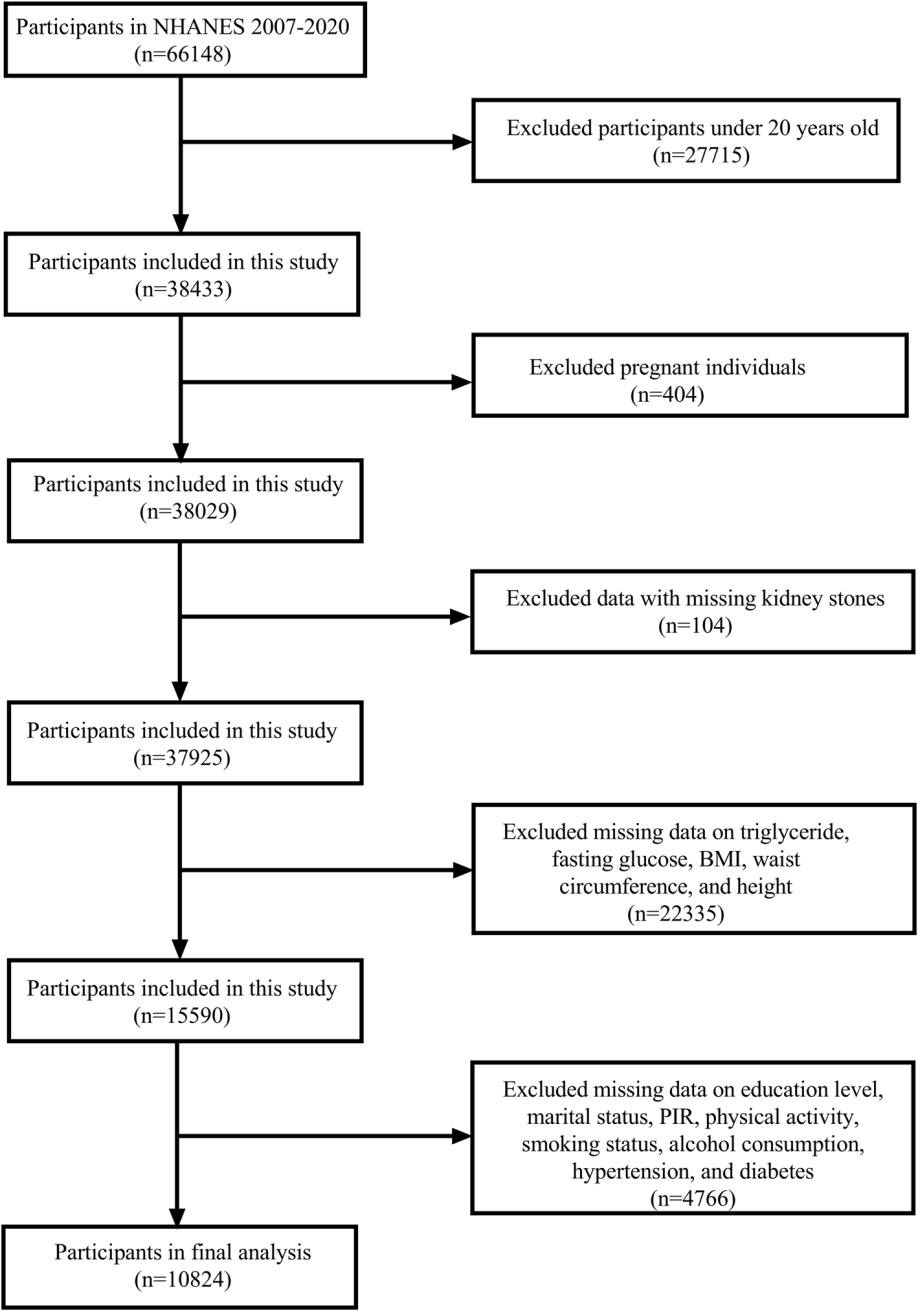
Flowchart of study participants selection from NHANES 2007-2020.

The NHANES cycles from 2007 to 2020 were selected for this study as they contained the necessary variables for our analysis. A total of 66,148 participants were included in these cycles. We first excluded participants younger than 20 years (n = 27,715), followed by the exclusion of pregnant individuals (n = 404) and those with missing data on kidney stone status (n = 104). Participants with incomplete data for triglycerides, fasting glucose, BMI, waist circumference (WC), and height (n = 22,335) were also excluded. Additionally, participants missing data on education, marital status, poverty-income ratio (PIR), physical activity, smoking habits, alcohol consumption, hypertension, and diabetes (n = 4,766) were excluded. After applying these criteria, the final analytical sample consisted of 10,824 participants (**Figure 1**).

### 2.3 Definitions of TyG−related indices

In this study, TyG-related indices were calculated using fasting triglyceride, fasting glucose levels, and body measurements. BMI was calculated as weight (in kilograms) divided by the square of height (in meters). The waist-to-height ratio (WHtR) was defined by dividing WC by height (17). The TyG index was calculated as follows: TyG index = ln [fasting triglyceride (mg/dL) × fasting glucose (mg/dL)/2] (18–20). Based on this, the following TyG-related indices were calculated: TyG-BMI = TyG × BMI (11, 21); TyG-WC = TyG × WC (21); TyG-WHtR = TyG × WHtR (22).

### 2.4 Kidney stones

In this study, the presence of kidney stones was determined based on responses to the NHANES questionnaire. Participants were classified as having a history of kidney stones if they answered “yes” to the question, “KIQ026 (Have you/Has the sample person ever had kidney stones)?”. Those who answered “no” were considered not to have a history of kidney stones.

### 2.5 Covariates

Covariates were selected based on their potential to influence the effect estimate by more than 10%, as well as their relevance from both clinical and literature perspectives (23–27). These covariates included age, gender, race, education level, marital status, PIR, physical activity, smoking status, alcohol consumption, hypertension, and diabetes. Age was analyzed both as a continuous variable (in years) and as a categorical variable with three groups: <40 years, 40–60 years, and ≥60 years. Gender was categorized as male or female. Race was divided into five groups: Mexican American, Other Hispanic, Non-Hispanic White, Non-Hispanic Black, and Other Race. Education level was classified into three categories: less than high school, high school or equivalent, and college or above. Marital status was classified into two categories: “married or living with a partner” and “single”, with “single” including participants who were never married, separated, divorced, or widowed. PIR, an indicator of socioeconomic status, was grouped into low (PIR < 1.3), medium (PIR 1.3–3.5), and high (PIR ≥ 3.5) (28).

Physical activity level was categorized according to the American Physical Activity Council’s recommendations for chronic health conditions (≥75 minutes per week of vigorous activity or ≥150 minutes per week of moderate activity). Participants were divided into three groups: active (meeting or exceeding the recommended level), less active (below the recommended level), and inactive (no physical activity) (29). Smoking status was categorized into never, former, and current smokers. Never smokers were defined as those who had smoked fewer than 100 cigarettes in their lifetime, former smokers had smoked more than 100 but had quit, and current smokers had smoked over 100 cigarettes and were still smoking (30, 31). Alcohol consumption was divided into four groups: none, moderate (up to 1 drink per day for women or 1–2 drinks per day for men), heavy (2–3 drinks per day for women or 3–4 drinks per day for men), and binge (≥4 drinks per day for women or ≥5 drinks per day for men), based on guidelines from the National Institute on Alcohol Abuse and Alcoholism (NIAAA). Hypertension was identified through self-reported high blood pressure, use of antihypertensive medication, or blood pressure readings of systolic BP ≥130 mmHg and/or diastolic BP ≥80 mmHg (32). Diabetes was defined as a self-reported diagnosis, use of diabetes medication, or insulin therapy.

### 2.6 Statistical Analysis

To account for the complex survey design and ensure that the data accurately represented the national population, survey weights from NHANES, including WTMEC2YR (full sample 2-year MEC exam weight), SDMVPSU (masked variance pseudo-PSU), and SDMVSTRA (masked variance pseudo-stratum), were applied. Baseline characteristics were analyzed using survey-weighted linear regression for continuous variables, while the chi-square test was used for categorical variables. Continuous variables were expressed as survey-weighted means ± standard error (SE), and categorical variables were presented as unweighted counts with percentages. Participants were grouped into tertiles based on TyG-BMI, TyG-WC, and TyG-WHtR values.

To explore the association between TyG-related indices and kidney stones, multivariate weighted logistic regression models were applied, with odds ratios (ORs) and 95% confidence intervals (CIs) reported. TyG-related indices were initially introduced as continuous variables and subsequently divided into tertiles for further analysis. Three models were constructed: Model 1 was unadjusted, Model 2 was adjusted for age, gender, and race, and Model 3 was further adjusted for additional covariates, including marital status, PIR, physical activity, smoking status, alcohol consumption, hypertension, and diabetes. Fully adjusted models with smooth curve fitting were applied to evaluate the potential non-linear relationship between TyG-related indices and kidney stones. Subgroup analyses were performed across various demographics and health conditions, such as age, gender, BMI, smoking status, alcohol consumption, hypertension, and diabetes, using multivariate weighted logistic regression models, adjusting for covariates similar to those in Model 3. Interaction terms were included to assess differences across subgroups. Receiver operating characteristic (ROC) curves were generated to compare the predictive accuracy of the three TyG indices, and cutoff values with corresponding sensitivity and specificity were identified. All analyses were conducted using Empower Stats (http://www.empowerstats.com, X&Y Solutions, Inc, CA, USA) and R software (version 4.4.0; https://www.R-project.org), with a two-sided P-value < 0.05 considered statistically significant.

## 3 Results

### 3.1 Characteristics of the participants

A total of 10,824 participants were included in the analysis. We classified them based on whether or not they had a history of kidney stones, identifying 986 participants with kidney stones. Participants with kidney stones tended to be older, with higher average BMI, greater WC, and an increased WHtR compared to those without kidney stones. A higher proportion of kidney stone patients were former smokers. Clinically, participants with kidney stones had a higher prevalence of hypertension and diabetes, as well as elevated fasting glucose and triglyceride levels. All TyG-related indices, including TyG-BMI, TyG-WC, and TyG-WHtR, were significantly higher in the kidney stone group compared to participants without stones (**Table 1**).

**Table 1:** Comparison of characteristics of participants with and without kidney stones.

	Total	No-kidney stones	Kidney stones	*P*-value
Number	10824	9838	986	
Gender				<0.001
Male	5586 (51.61%)	5024 (51.07%)	562 (57.00%)	
Female	5238 (48.39%)	4814 (48.93%)	424 (43.00%)	
Age (years)	46.69 ± 0.3	46.13 ± 0.31	52.10 ± 0.64	<0.001
Race, n (%)				<0.001
Mexican American	1599 (14.77%)	1466 (14.90%)	133 (13.49%)	
Other Hispanic	1084 (10.01%)	967 (9.83%)	117 (11.87%)	
Non-Hispanic White	4756 (43.94%)	4216 (42.85%)	540 (54.77%)	
Non-Hispanic Black	2165 (20.00%)	2057 (20.91%)	108 (10.95%)	
Other Race - Including Multi-Racial	1220 (11.27%)	1132 (11.51%)	88 (8.92%)	
Education level, n (%)				0.903
Less than high school	2264 (20.92%)	2062 (20.96%)	202 (20.49%)	
High school or equivalent	2440 (22.54%)	2220 (22.57%)	220 (22.31%)	
College or above	6120 (56.54%)	5556 (56.47%)	564 (57.20%)	
PIR, n (%)				0.624
< 1.3	3207 (29.63%)	2928 (29.76%)	279 (28.30%)	
1.3-3.5	4065 (37.56%)	3686 (37.47%)	379 (38.44%)	
≥ 3.5	3552 (32.82%)	3224 (32.77%)	328 (33.27%)	
Physical activity level, n (%)				0.790
Inactive	5972 (55.17%)	5427 (55.16%)	545 (55.27%)	
Less active	845 (7.81%)	763 (7.76%)	82 (8.32%)	
Active	4007 (37.02%)	3648 (37.08%)	359 (36.41%)	
Marital status, n (%)				<0.001
Single	4320 (39.91%)	3990 (40.56%)	330 (33.47%)	
Married or living with partner	6504 (60.09%)	5848 (59.44%)	656 (66.53%)	
Smoking status, n (%)				0.008
Never	5867 (54.20%)	5361 (54.49%)	506 (51.32%)	
Former	2657 (24.55%)	2375 (24.14%)	282 (28.60%)	
Current	2300 (21.25%)	2102 (21.37%)	198 (20.08%)	
Alcohol consumption, n (%)				0.041
None	1344 (12.42%)	1219 (12.39%)	125 (12.68%)	
Moderate	3952 (36.51%)	3576 (36.35%)	376 (38.13%)	
Heavy	2559 (23.64%)	2362 (24.01%)	197 (19.98%)	
Binge	2969 (27.43%)	2681 (27.25%)	288 (29.21%)	
Hypertension				<0.001
No	5348 (49.41%)	4974 (50.56%)	374 (37.93%)	
Yes	5476 (50.59%)	4864 (49.44%)	612 (62.07%)	
Diabetes				<0.001
No	9439 (87.20%)	8675 (88.18%)	764 (77.48%)	
Yes	1385 (12.80%)	1163 (11.82%)	222 (22.52%)	
Weight (kg)	83.32 ± 0.29	82.75 ± 0.29	88.69 ± 0.91	<0.001
Height (cm)	169.44 ± 0.13	169.47 ± 0.14	169.13 ± 0.45	0.461
BMI (kg/m2)	28.93 ± 0.09	28.73 ± 0.09	30.92 ± 0.30	<0.001
WC (cm)	99.21 ± 0.25	98.59 ± 0.25	105.18 ± 0.66	<0.001
WHtR	0.59 ± 0.00	0.58 ± 0.00	0.62 ± 0.00	<0.001
HDL-C (mg/dL)	54.4 ± 0.29	54.77 ± 0.32	50.81 ± 0.60	<0.001
Fasting glucose (mg/dL)	106.07 ± 0.41	105.47 ± 0.43	111.82 ± 1.07	<0.001
Triglyceride (mg/dL)	121.47 ± 1.37	120.29 ± 1.51	132.73 ± 3.73	0.004
TyG	8.56 ± 0.01	8.54 ± 0.01	8.72 ± 0.03	<0.001
TyG-BMI	249.05 ± 1.00	246.77 ± 1.02	270.80 ± 2.92	<0.001
TyG-WC	853.47 ± 2.79	846.44 ± 2.87	920.62 ± 6.90	<0.001
TyG-WHtR	5.04 ± 0.02	5.00 ± 0.02	5.45 ± 0.04	<0.001

Continuous variables were presented as survey-weighted means ± standard error (SE), with *p*-values determined using survey-weighted linear regression. Categorical variables were reported as unweighted counts (percentages), and *p*-values were obtained through the chi-square test.

PIR, Poverty-income ratio; HDL-C, high‐density lipoprotein cholesterol; WC, waist circumference; WHtR, waist-height ratio; TyG, triglyceride glucose index; TyG-BMI, triglyceride glucose-body mass index; TyG-WC, triglyceride glucose-waist circumference; TyG-WHtR, triglyceride glucose-waist to height ratio.

Participants were then categorized into tertiles based on TyG-BMI, TyG-WC, and TyG-WHtR, and their characteristics were compared across these groups (**Tables 2**–**4**). Analysis revealed that participants in the highest tertiles for all TyG-related indices (TyG-BMI, TyG-WC, and TyG-WHtR) were consistently older and had higher BMI, WHtR, WC, fasting glucose, and triglyceride levels compared to those in the lower tertiles. Furthermore, participants in the highest tertile exhibited lower HDL-C levels, lower levels of education, and a higher likelihood of smoking and alcohol consumption. The prevalence of both hypertension and diabetes was also significantly higher in in the highest tertile group.

**Table 2:** Baseline characteristics according to TyG-BMI tertiles.

TyG-BMI	Tertile 1 (113.51-215.95)	Tertile 2 (215.96-268.49)	Tertile 3 (268.49-679.46)	*P*-value
Kidney stones				<0.001
No	3392 (94.01%)	3276 (90.80%)	3170 (87.86%)	
Yes	216 (5.99%)	332 (9.20%)	438 (12.14%)	
Gender				<0.001
Male	1713 (47.48%)	2080 (57.65%)	1793 (49.70%)	
Female	1895 (52.52%)	1528 (42.35%)	1815 (50.30%)	
Age (years)	43.39 ± 0.52	48.86 ± 0.37	47.99 ± 0.42	<0.001
Race, n (%)				<0.001
Mexican American	317 (8.79%)	608 (16.85%)	674 (18.68%)	
Other Hispanic	288 (7.98%)	406 (11.25%)	390 (10.81%)	
Non-Hispanic White	1650 (45.73%)	1554 (43.07%)	1552 (43.02%)	
Non-Hispanic Black	719 (19.93%)	671 (18.60%)	775 (21.48%)	
Other Race - Including Multi-Racial	634 (17.57%)	369 (10.23%)	217 (6.01%)	
Education level, n (%)				<0.001
Less than high school	616 (17.07%)	815 (22.59%)	833 (23.09%)	
High school or equivalent	752 (20.84%)	824 (22.84%)	864 (23.95%)	
College or above	2240 (62.08%)	1969 (54.57%)	1911 (52.97%)	
PIR, n (%)				<0.001
< 1.3	1017 (28.19%)	1009 (27.97%)	1181 (32.73%)	
1.3-3.5	1285 (35.62%)	1372 (38.03%)	1408 (39.02%)	
≥ 3.5	1306 (36.20%)	1227 (34.01%)	1019 (28.24%)	
Physical activity level, n (%)				0.198
Inactive	2036 (56.43%)	1973 (54.68%)	1963 (54.41%)	
Less active	281 (7.79%)	297 (8.23%)	267 (7.40%)	
Active	1291 (35.78%)	1338 (37.08%)	1378 (38.19%)	
Marital status, n (%)				<0.001
Single	1598 (44.29%)	1319 (36.56%)	1403 (38.89%)	
Married or living with partner	2010 (55.71%)	2289 (63.44%)	2205 (61.11%)	
Smoking status, n (%)				<0.001
Never	2038 (56.49%)	1919 (53.19%)	1910 (52.94%)	
Former	727 (20.15%)	931 (25.80%)	999 (27.69%)	
Current	843 (23.36%)	758 (21.01%)	699 (19.37%)	
Alcohol consumption, n (%)				<0.001
None	439 (12.17%)	427 (11.83%)	478 (13.25%)	
Moderate	1414 (39.19%)	1352 (37.47%)	1186 (32.87%)	
Heavy	890 (24.67%)	843 (23.36%)	826 (22.89%)	
Binge	865 (23.97%)	986 (27.33%)	1118 (30.99%)	
Hypertension				<0.001
No	2375 (65.83%)	1736 (48.12%)	1237 (34.28%)	
Yes	1233 (34.17%)	1872 (51.88%)	2371 (65.72%)	
Diabetes				<0.001
No	3446 (95.51%)	3211 (89.00%)	2782 (77.11%)	
Yes	162 (4.49%)	397 (11.00%)	826 (22.89%)	
Weight (kg)	65.22 ± 0.26	81.64 ± 0.23	104.05 ± 0.42	<0.001
Height (cm)	168.81 ± 0.23	170.25 ± 0.21	169.27 ± 0.24	<0.001
BMI (kg/m2)	22.8 ± 0.06	28.08 ± 0.06	36.26 ± 0.13	<0.001
WC (cm)	83.82 ± 0.19	98.49 ± 0.19	116.13 ± 0.31	<0.001
WHtR	0.50 ± 0.00	0.58 ± 0.00	0.69 ± 0.00	<0.001
HDL-C (mg/dL)	63.61 ± 0.53	52.86 ± 0.35	46.25 ± 0.28	<0.001
Fasting glucose (mg/dL)	96.72 ± 0.31	104.72 ± 0.64	117.28 ± 0.84	<0.001
Triglyceride (mg/dL)	77.68 ± 0.92	120.05 ± 1.72	168.99 ± 3.08	<0.001
TyG	8.11 ± 0.01	8.6 ± 0.01	8.99 ± 0.02	<0.001

Continuous variables were presented as survey-weighted means ± standard error (SE), with *p*-values determined using survey-weighted linear regression. Categorical variables were reported as unweighted counts (percentages), and *p*-values were obtained through the chi-square test.

PIR, Poverty-income ratio; HDL-C, high‐density lipoprotein cholesterol; WC, waist circumference; WHtR, waist-height ratio; TyG, triglyceride glucose index; TyG-BMI, triglyceride glucose-body mass index; TyG-WC, triglyceride glucose-waist circumference; TyG-WHtR, triglyceride glucose-waist to height ratio.

**Table 3:** Baseline characteristics according to TyG-WC tertiles.

TyG-WC	Tertile 1 (453.12-768.24)	Tertile 2 (768.27-918.67)	Tertile 3 (918.68-1697.36)	*P*-value
Kidney stones				<0.001
No	3415 (94.65%)	3278 (90.85%)	3145 (87.17%)	
Yes	193 (5.35%)	330 (9.15%)	463 (12.83%)	
Gender				<0.001
Male	1461 (40.49%)	1999 (55.40%)	2126 (58.92%)	
Female	2147 (59.51%)	1609 (44.60%)	1482 (41.08%)	
Age (years)	41.10 ± 0.49	48.8 ± 0.36	50.38 ± 0.40	<0.001
Race, n (%)				<0.001
Mexican American	367 (10.17%)	605 (16.77%)	627 (17.38%)	
Other Hispanic	317 (8.79%)	398 (11.03%)	369 (10.23%)	
Non-Hispanic White	1493 (41.38%)	1536 (42.57%)	1727 (47.87%)	
Non-Hispanic Black	810 (22.45%)	677 (18.76%)	678 (18.79%)	
Other Race - Including Multi-Racial	621 (17.21%)	392 (10.86%)	207 (5.74%)	
Education level, n (%)				<0.001
Less than high school	586 (16.24%)	830 (23.00%)	848 (23.50%)	
High school or equivalent	739 (20.48%)	822 (22.78%)	879 (24.36%)	
College or above	2283 (63.28%)	1956 (54.21%)	1881 (52.13%)	
PIR, n (%)				<0.001
< 1.3	1031 (28.58%)	1027 (28.46%)	1149 (31.85%)	
1.3-3.5	1299 (36.00%)	1379 (38.22%)	1387 (38.44%)	
≥ 3.5	1278 (35.42%)	1202 (33.31%)	1072 (29.71%)	
Physical activity level, n (%)				0.288
Inactive	2026 (56.15%)	2007 (55.63%)	1939 (53.74%)	
Less active	269 (7.46%)	284 (7.87%)	292 (8.09%)	
Active	1313 (36.39%)	1317 (36.50%)	1377 (38.17%)	
Marital status, n (%)				<0.001
Single	1655 (45.87%)	1288 (35.70%)	1377 (38.17%)	
Married or living with partner	1953 (54.13%)	2320 (64.30%)	2231 (61.83%)	
Smoking status, n (%)				<0.001
Never	2185 (60.56%)	1931 (53.52%)	1751 (48.53%)	
Former	627 (17.38%)	900 (24.94%)	1130 (31.32%)	
Current	796 (22.06%)	777 (21.54%)	727 (20.15%)	
Alcohol consumption, n (%)				<0.001
None	456 (12.64%)	442 (12.25%)	446 (12.36%)	
Moderate	1361 (37.72%)	1359 (37.67%)	1232 (34.15%)	
Heavy	1000 (27.72%)	812 (22.51%)	747 (20.70%)	
Binge	791 (21.92%)	995 (27.58%)	1183 (32.79%)	
Hypertension				<0.001
No	2535 (70.26%)	1697 (47.03%)	1116 (30.93%)	
Yes	1073 (29.74%)	1911 (52.97%)	2492 (69.07%)	
Diabetes				<0.001
No	3495 (96.87%)	3248 (90.02%)	2696 (74.72%)	
Yes	113 (3.13%)	360 (9.98%)	912 (25.28%)	
Weight (kg)	65.51 ± 0.27	81.55 ± 0.28	103.46 ± 0.43	<0.001
Height (cm)	167.4 ± 0.24	169.6 ± 0.22	171.38 ± 0.25	<0.001
BMI (kg/m2)	23.34 ± 0.08	28.37 ± 0.09	35.28 ± 0.14	<0.001
WC (cm)	83.13 ± 0.18	98.48 ± 0.17	116.54 ± 0.31	<0.001
WHtR	0.50 ± 0.00	0.58 ± 0.00	0.68 ± 0.00	<0.001
HDL-C (mg/dL)	63.97 ± 0.5	53.56 ± 0.38	45.35 ± 0.26	<0.001
Fasting glucose (mg/dL)	95.51 ± 0.31	103.11 ± 0.39	119.93 ± 0.93	<0.001
Triglyceride (mg/dL)	73.78 ± 0.91	114.27 ± 1.24	177.88 ± 2.94	<0.001
TyG	8.06 ± 0.01	8.57 ± 0.01	9.07 ± 0.02	<0.001

Continuous variables were presented as survey-weighted means ± standard error (SE), with *p*-values determined using survey-weighted linear regression. Categorical variables were reported as unweighted counts (percentages), and *p*-values were obtained through the chi-square test.

PIR, Poverty-income ratio; HDL-C, high‐density lipoprotein cholesterol; WC, waist circumference; WHtR, waist-height ratio; TyG, triglyceride glucose index; TyG-BMI, triglyceride glucose-body mass index; TyG-WC, triglyceride glucose-waist circumference; TyG-WHtR, triglyceride glucose-waist to height ratio.

**Table 4:** Baseline characteristics according to TyG-WHtR tertiles.

TyG-WHtR	Tertile 1 (2.58-4.57)	Tertile 2 (4.57-5.47)	Tertile 3 (5.47-10.64)	*P*-value
Kidney stones				<0.001
No	3404 (94.35%)	3274 (90.74%)	3160 (87.58%)	
Yes	204 (5.65%)	334 (9.26%)	448 (12.42%)	
Gender				<0.001
Male	1833 (50.80%)	2054 (56.93%)	1699 (47.09%)	
Female	1775 (49.20%)	1554 (43.07%)	1909 (52.91%)	
Age (years)	40.53 ± 0.46	48.99 ± 0.35	51.25 ± 0.39	<0.001
Race, n (%)				<0.001
Mexican American	315 (8.73%)	581 (16.10%)	703 (19.48%)	
Other Hispanic	299 (8.29%)	355 (9.84%)	430 (11.92%)	
Non-Hispanic White	1580 (43.79%)	1573 (43.60%)	1603 (44.43%)	
Non-Hispanic Black	837 (23.20%)	682 (18.90%)	646 (17.90%)	
Other Race - Including Multi-Racial	577 (15.99%)	417 (11.56%)	226 (6.26%)	
Education level, n (%)				<0.001
Less than high school	537 (14.88%)	782 (21.67%)	945 (26.19%)	
High school or equivalent	737 (20.43%)	845 (23.42%)	858 (23.78%)	
College or above	2334 (64.69%)	1981 (54.91%)	1805 (50.03%)	
PIR, n (%)				<0.001
< 1.3	985 (27.30%)	999 (27.69%)	1223 (33.90%)	
1.3-3.5	1288 (35.70%)	1369 (37.94%)	1408 (39.02%)	
≥ 3.5	1335 (37.00%)	1240 (34.37%)	977 (27.08%)	
Physical activity level, n (%)				0.044
Inactive	1934 (53.60%)	1975 (54.74%)	2063 (57.18%)	
Less active	293 (8.12%)	281 (7.79%)	271 (7.51%)	
Active	1381 (38.28%)	1352 (37.47%)	1274 (35.31%)	
Marital status, n (%)				<0.001
Single	1629 (45.15%)	1277 (35.39%)	1414 (39.19%)	
Married or living with partner	1979 (54.85%)	2331 (64.61%)	2194 (60.81%)	
Smoking status, n (%)				<0.001
Never	2101 (58.23%)	1899 (52.63%)	1867 (51.75%)	
Former	666 (18.46%)	934 (25.89%)	1057 (29.30%)	
Current	841 (23.31%)	775 (21.48%)	684 (18.96%)	
Alcohol consumption, n (%)				<0.001
None	369 (10.23%)	436 (12.08%)	539 (14.94%)	
Moderate	1412 (39.14%)	1342 (37.20%)	1198 (33.20%)	
Heavy	951 (26.36%)	844 (23.39%)	764 (21.18%)	
Binge	876 (24.28%)	986 (27.33%)	1107 (30.68%)	
Hypertension				<0.001
No	2540 (70.40%)	1688 (46.78%)	1120 (31.04%)	
Yes	1068 (29.60%)	1920 (53.22%)	2488 (68.96%)	
Diabetes				<0.001
No	3511 (97.31%)	3264 (90.47%)	2664 (73.84%)	
Yes	97 (2.69%)	344 (9.53%)	944 (26.16%)	
Weight (kg)	68.37 ± 0.33	82.38 ± 0.31	101.4 ± 0.48	<0.001
Height (cm)	170.4 ± 0.24	169.74 ± 0.2	168.01 ± 0.25	<0.001
BMI (kg/m2)	23.41 ± 0.08	28.45 ± 0.08	35.77 ± 0.14	<0.001
WC (cm)	84.29 ± 0.21	99 ± 0.17	116.48 ± 0.31	<0.001
WHtR	0.49 ± 0.00	0.58 ± 0.00	0.69 ± 0.00	<0.001
HDL-C (mg/dL)	62.48 ± 0.48	52.88 ± 0.4	46.83 ± 0.31	<0.001
Fasting glucose (mg/dL)	95.77 ± 0.30	103.78 ± 0.55	120.32 ± 0.91	<0.001
Triglyceride (mg/dL)	74.52 ± 0.93	120.05 ± 1.53	176.64 ± 3.23	<0.001
TyG	8.07 ± 0.01	8.61 ± 0.01	9.06 ± 0.02	<0.001

Continuous variables were presented as survey-weighted means ± standard error (SE), with *p*-values determined using survey-weighted linear regression. Categorical variables were reported as unweighted counts (percentages), and *p*-values were obtained through the chi-square test.

PIR, Poverty-income ratio; HDL-C, high‐density lipoprotein cholesterol; WC, waist circumference; WHtR, waist-height ratio; TyG, triglyceride glucose index; TyG-BMI, triglyceride glucose-body mass index; TyG-WC, triglyceride glucose-waist circumference; TyG-WHtR, triglyceride glucose-waist to height ratio.

### 3.2 Association between TyG-related indices and kidney stones

**Figure 2:**
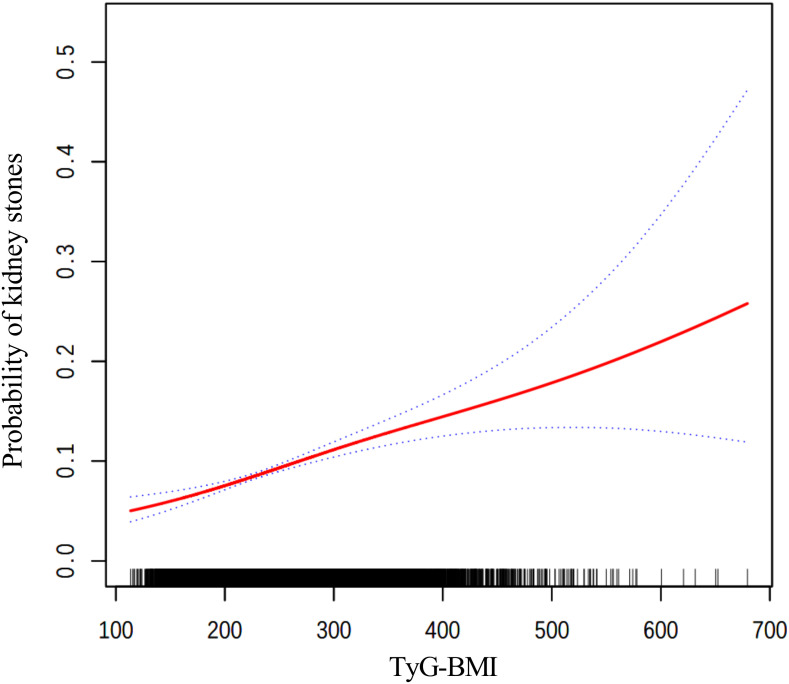
The smooth curve fitting between TyG-BMI and kidney stones. Adjusted for: age, gender, race, marital status, poverty-income ratio, physical activity, smoking status, alcohol consumption, hypertension, and diabetes.

**Figure 3:**
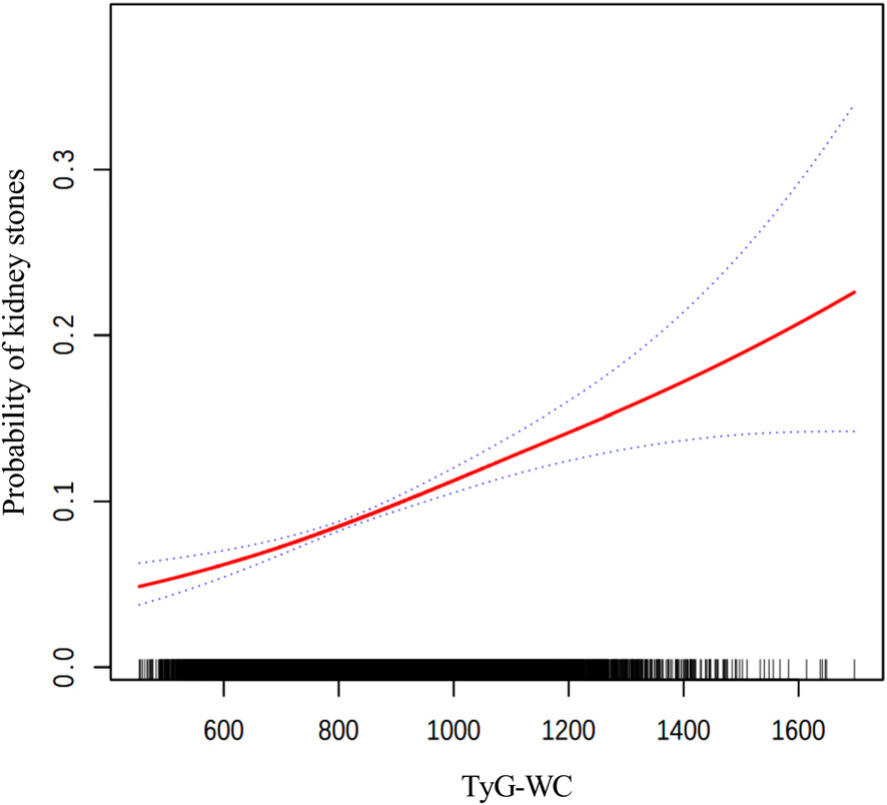
The smooth curve fitting between TyG-WC and kidney stones. Adjusted for: age, gender, race, marital status, poverty-income ratio, physical activity, smoking status, alcohol consumption, hypertension, and diabetes.

**Figure 4:**
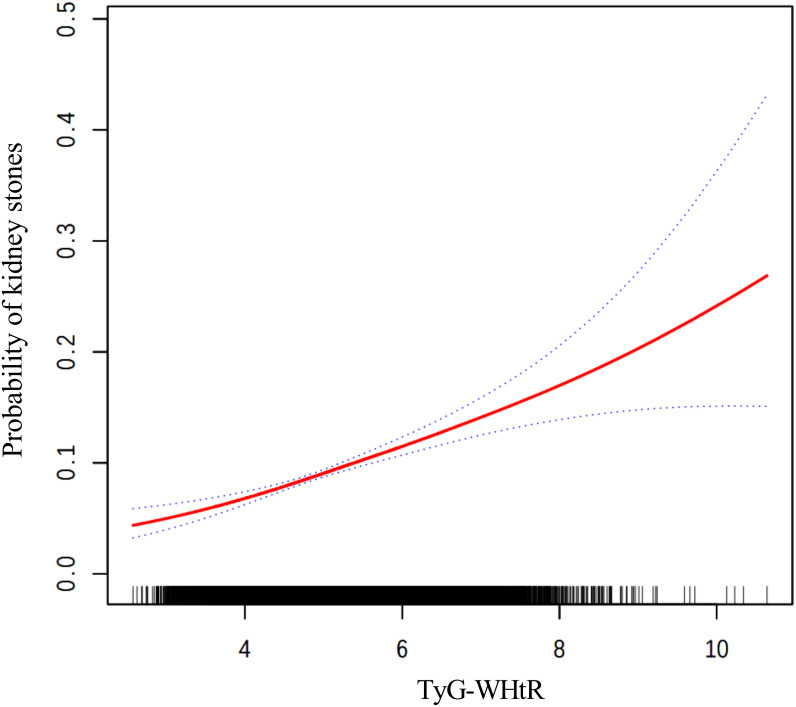
The smooth curve fitting between TyG-WHtR and kidney stones. Adjusted for: age, gender, race, marital status, poverty-income ratio, physical activity, smoking status, alcohol consumption, hypertension, and diabetes.

The findings from the weighted logistic regression analyses are presented in **Table 5**. TyG-related indices (TyG-BMI, TyG-WC, TyG-WHtR) were positively associated with the risk of kidney stones in all models. As continuous variables, each unit increase in TyG-BMI, TyG-WC, and TyG-WHtR was associated with a higher risk of kidney stones, with statistically significant results across all three models (*P* < 0.001). In the fully adjusted model, the OR for TyG-BMI was 1.0040 (95% CI: 1.0028, 1.0052), for TyG-WC 1.0015 (95% CI: 1.0011, 1.0020), and for TyG-WHtR 1.3305 (95% CI: 1.2277, 1.4419). When dividing participants into tertiles, the trend remained consistent. For TyG-BMI, those in the highest tertile had an OR of 1.9924 (95% CI: 1.5704, 2.5277) in Model 3 compared to the reference group in the lowest tertile. A similar pattern was observed for TyG-WC and TyG-WHtR. In TyG-WC tertiles, participants in tertile 3 had an OR of 2.0303 (95% CI: 1.5622, 2.6388) in Model 3. For TyG-WHtR, tertile 3 participants had an OR of 1.7076 (95% CI: 1.2663, 2.3028) in the fully adjusted model. The p-values for the trend across tertiles were consistently significant (*P* for trend < 0.001). The smooth curve fitting analysis (**Figures 2**–**4**) showed a positive association between TyG-BMI, TyG-WC, and TyG-WHtR and the probability of kidney stones. As each TyG-related index increased, the probability of kidney stones rose consistently across all models.

**Table 5:** Association between TyG-BMI, TyG-WC, TyG-WHtR and kidney stones.

	Model 1 OR (95%CI), *P* value	Model 2 OR (95%CI), *P* value	Model 3 OR (95%CI), *P* value
**TyG-BMI (continuous)**	1.0048 (1.0038, 1.0059) <0.001	1.0049 (1.0038, 1.0060) <0.001	1.0040 (1.0028, 1.0052) <0.001
TyG-BMI (tertiles)			
Tertile 1	Reference	Reference	Reference
Tertile 2	1.7535 (1.4074, 2.1848) <0.001	1.5637 (1.2412, 1.9700) <0.001	1.4970 (1.1769, 1.9041) 0.002
Tertile 3	2.4350 (1.9624, 3.0213) <0.001	2.2831 (1.8278, 2.8517) <0.001	1.9924 (1.5704, 2.5277) <0.001
P for trend	<0.001	<0.001	<0.001
**TyG-WC (continuous)**	1.0022 (1.0018, 1.0026) <0.001	1.0019 (1.0014, 1.0023) <0.001	1.0015 (1.0011, 1.0020) <0.001
TyG-WC (tertiles)			
Tertile 1	Reference	Reference	Reference
Tertile 2	1.8650 (1.4914, 2.3323) <0.001	1.6117 (1.2798, 2.0298) 0.001	1.5245 (1.2075, 1.9247) <0.001
Tertile 3	2.8006 (2.2451, 3.4936) <0.001	2.3560 (1.8544, 2.9932) <0.001	2.0303 (1.5622, 2.6388) <0.001
P for trend	<0.001	<0.001	<0.001
**TyG-WHtR (continuous)**	1.4650 (1.3684, 1.5685) <0.001	1.4062 (1.3071, 1.5129) <0.001	1.3305 (1.2277, 1.4419) <0.001
TyG-WHtR (tertile)			
Tertile 1	Reference	Reference	Reference
Tertile 2	1.6640 (1.2841, 2.1564) <0.001	1.4109 (1.0694, 1.8614) 0.017	1.3356 (0.9922, 1.7978) 0.060
Tertile 3	2.4033 (1.8726, 3.0845) <0.001	2.0152 (1.5433, 2.6315) <0.001	1.7076 (1.2663, 2.3028) <0.001
P for trend	<0.001	<0.001	<0.001

Model 1: non-adjusted model.

Model 2: adjusted for: age, gender, and race.

Model 3: adjusted for: age, gender, race, marital status, poverty-income ratio, physical activity, smoking status, alcohol consumption, hypertension, and diabetes.

TyG-BMI, triglyceride glucose-body mass index; TyG-WC, triglyceride glucose-waist circumference; TyG-WHtR, triglyceride glucose-waist to height ratio.

### 3.3 Subgroup analysis

The subgroup analysis results presented in **Table 6** indicate that the associations between TyG-BMI, TyG-WC, and TyG-WHtR with kidney stones were statistically significant across most subgroups, including gender, age, BMI, smoking status, alcohol consumption, hypertension, and diabetes (*P* < 0.05). The associations remained largely consistent without significant interaction effects for most variables (*P* for interaction > 0.05). However, a significant interaction was observed between alcohol consumption and TyG-WHtR (*P* for interaction = 0.045).

**Table 6:** Subgroup analysis for the associations of TyG-related indices with kidney stones.

	OR (95%CI)	*P*-value	*P* for interaction
TyG-BMI			
Gender			0.809
Male	1.0042 (1.0025, 1.0059)	<0.001	
Female	1.0039 (1.0022, 1.0055)	<0.001	
Age			0.931
< 40	1.0036 (1.0016, 1.0055)	0.001	
40-60	1.0039 (1.0019, 1.0059)	<0.001	
≥ 60	1.0041 (1.0019, 1.0062)	<0.001	
BMI			0.791
< 25	1.0058 (0.9956, 1.0161)	0.268	
25-30	1.0024 (0.9966, 1.0083)	0.418	
≥ 30	1.0021 (0.9996, 1.0046)	0.100	
Smoking status			0.480
Never	1.0045 (1.0030, 1.0059)	<0.001	
Former	1.0038 (1.0013, 1.0064)	0.004	
Current	1.0029 (1.0004, 1.0054)	0.026	
Alcohol consumption			0.118
None	1.0059 (1.0032, 1.0085)	<0.001	
Moderate	1.0032 (1.0014, 1.0050)	0.001	
Heavy	1.0057 (1.0031, 1.0083)	<0.001	
Binge	1.0029 (1.0007, 1.0051)	0.010	
Hypertension			0.214
No	1.0050 (1.0031, 1.0068)	<0.001	
Yes	1.0034 (1.0018, 1.0049)	<0.001	
Diabetes			0.451
No	1.0037 (1.0023, 1.0052)	<0.001	
Yes	1.0050 (1.0023, 1.0076)	<0.001	
TyG-WC			
Gender			0.978
Male	1.0015 (1.0009, 1.0022)	<0.001	
Female	1.0015 (1.0008, 1.0022)	<0.001	
Age			0.896
< 40	1.0014 (1.0006, 1.0021)	0.001	
40-60	1.0015 (1.0008, 1.0023)	<0.001	
≥ 60	1.0016 (1.0008, 1.0025)	<0.001	
BMI			0.720
< 25	1.0016 (0.9990, 1.0041)	0.237	
25-30	1.0012 (0.9997, 1.0026)	0.111	
≥ 30	1.0006 (0.9997, 1.0015)	0.200	
Smoking status			0.252
Never	1.0018 (1.0013, 1.0024)	<0.001	
Former	1.0013 (1.0003, 1.0023)	0.013	
Current	1.0010 (1.0000, 1.0020)	0.043	
Alcohol consumption			0.079
None	1.0028 (1.0016, 1.0040)	<0.001	
Moderate	1.0012 (1.0005, 1.0019)	0.001	
Heavy	1.0021 (1.0011, 1.0030)	<0.001	
Binge	1.0012 (1.0003, 1.0020)	0.011	
Hypertension			0.082
No	1.0020 (1.0013, 1.0028)	<0.001	
Yes	1.0012 (1.0006, 1.0018)	<0.001	
Diabetes			0.440
No	1.0014 (1.0009, 1.0020)	<0.001	
Yes	1.0019 (1.0009, 1.0029)	<0.001	
TyG-WHtR			
Gender			0.765
Male	1.3475 (1.2083, 1.5028)	<0.001	
Female	1.3154 (1.1715, 1.4769)	<0.001	
Age			0.943
< 40	1.3431 (1.1812, 1.5271)	<0.001	
40-60	1.3035 (1.1412, 1.4888)	<0.001	
≥ 60	1.3365 (1.1584, 1.5418)	<0.001	
BMI			0.704
< 25	1.4307 (0.9228, 2.2180)	0.113	
25-30	1.2304 (0.9238, 1.6386)	0.160	
≥ 30	1.1773 (1.0067, 1.3767)	0.044	
Smoking status			0.553
Never	1.3804 (1.2470, 1.5280)	<0.001	
Former	1.2837 (1.0773, 1.5297)	0.007	
Current	1.2624 (1.0776, 1.4789)	0.005	
Alcohol consumption			0.045
None	1.6203 (1.3415, 1.9569)	<0.001	
Moderate	1.2487 (1.0988, 1.4191)	0.001	
Heavy	1.4782 (1.2581, 1.7367)	<0.001	
Binge	1.2353 (1.0725, 1.4227)	0.004	
Hypertension			0.068
No	1.4503 (1.2853, 1.6365)	<0.001	
Yes	1.2484 (1.1243, 1.3862)	<0.001	
Diabetes			0.413
No	1.3073 (1.1894, 1.4368)	<0.001	
Yes	1.4292 (1.1896, 1.7171)	<0.001	

Age, gender, race, marital status, poverty-income ratio, physical activity, smoking status, alcohol consumption, hypertension, and diabetes were all adjusted except the stratification variable.

BMI, body mass index; TyG-BMI, triglyceride glucose-body mass index; TyG-WC, triglyceride glucose-waist circumference; TyG-WHtR, triglyceride glucose-waist to height ratio.

### 3.4 Comparison of TyG-related indices in predicting kidney stones

**Figure 5:**
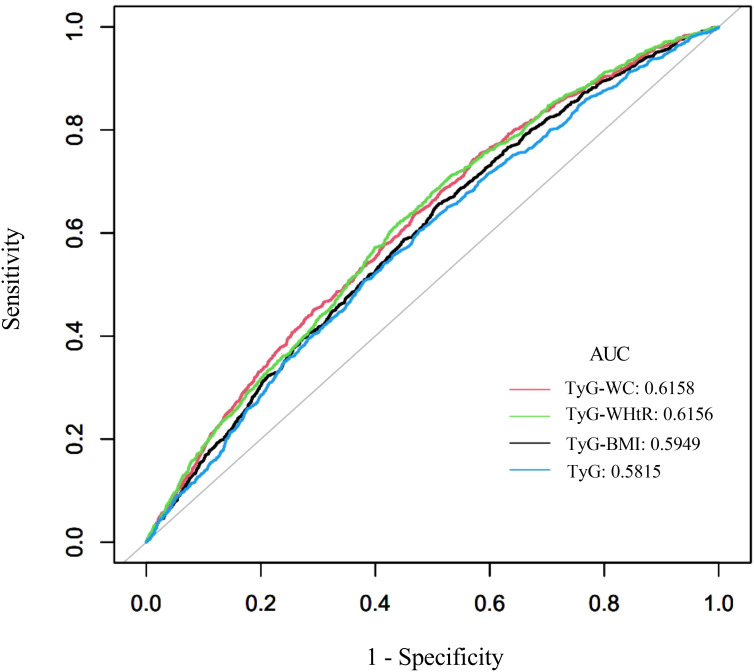
ROC curves of the TyG-related indices for predicting kidney stones.

The diagnostic performance of TyG-related indices (TyG, TyG-BMI, TyG-WC, and TyG-WHtR) in predicting kidney stones was assessed using area under the curve (AUC) values, as shown in **Figure 5** (ROC curves) and **Table 7**. Among all indices, TyG-WC demonstrated the highest predictive capability, with an AUC of 0.6158, closely followed by TyG-WHtR (AUC = 0.6156). TyG-BMI showed slightly lower predictive power (AUC = 0.5949), while TyG itself had the lowest AUC (0.5815). Sensitivity was highest for TyG-WC (0.7434), and specificity was greatest for TyG (0.5235). The cutoff values for the indices varied, with TyG-WC at 802.9051, TyG-WHtR at 4.8833, TyG-BMI at 236.6537, and TyG at 8.5577. These findings suggest that TyG-WC and TyG-WHtR have slightly better predictive performance than TyG and TyG-BMI.

**Table 7:** Performance assessment of the TyG and TyG-related indices in predicting kidney stones.

	AUC (95%CI)	Cutoff threshold	Sensitivity	Specificity
TyG	0.5815	8.5577	0.6045	0.5235
TyG-BMI	0.5949	236.6537	0.6572	0.4889
TyG-WC	0.6158	802.9051	0.7434	0.4282
TyG-WHtR	0.6156	4.8833	0.7130	0.4669

AUC, area under the curve; CI, confidence interval; TyG, triglyceride glucose index; TyG-BMI, triglyceride glucose-body mass index; TyG-WC, triglyceride glucose-waist circumference; TyG-WHtR, triglyceride glucose-waist to height ratio.

## 4 Discussion

This study investigated the associations between TyG-related indices (TyG-BMI, TyG-WC, and TyG-WHtR) and the risk of kidney stones using a large, nationally representative dataset. The findings demonstrated that higher levels of all three TyG-related indices were significantly associated with an increased likelihood of kidney stone formation. Among the indices, TyG-WC and TyG-WHtR exhibited the strongest predictive ability, as reflected by their higher AUC values compared to TyG-BMI and the TyG index alone. Additionally, the subgroup analysis indicated that these associations were consistent across various demographic and clinical subgroups, with no significant interactions observed for most variables, except for alcohol consumption, which showed a significant interaction with TyG-WHtR. These findings suggest that TyG-related indices, especially TyG-WC and TyG-WHtR, may serve as useful markers for assessing kidney stone risk in clinical practice.

The findings of our study align with previous research highlighting the positive association between the TyG index and its related indices with kidney stone formation. In earlier studies, Jiang et al (15). and Qin et al (16). demonstrated that elevated TyG index values are significantly associated with a higher risk of nephrolithiasis. Notably, as shown in **Table 1**, our findings revealed that participants with kidney stones exhibited characteristics consistent with metabolic syndrome. Specifically, the kidney stone group displayed significantly greater waist circumference, elevated fasting glucose levels, adverse lipid profiles (higher triglycerides and lower HDL-C), and a higher prevalence of hypertension and diabetes, all of which align with the established diagnostic criteria for metabolic syndrome (33, 34). This observed association between kidney stones and metabolic disturbances is consistent with findings from recent studies. These studies (14, 33–37) have demonstrated that metabolic syndrome increases kidney stone risk through various pathophysiological mechanisms, including altered urinary composition, heightened oxidative stress, and systemic inflammation. These findings underscore the importance of recognizing metabolic syndrome as a significant risk factor in the management of kidney stone disease. Furthermore, our study extends these findings by confirming the associations between kidney stones and TyG-related indices, particularly TyG-WC and TyG-WHtR, which combine anthropometric data with metabolic markers, potentially offering a more accurate reflection of metabolic dysfunction than the TyG index alone (10, 38).

Obesity, particularly severe obesity (BMI ≥35), is associated with increased morbidity and mortality, as well as various complications, including cardiovascular disease, type 2 diabetes mellitus, kidney stones, and others (39). Given these associations, we combined different obesity-related indices (BMI, WC, and WHtR) with the TyG index to evaluate their associations with kidney stones. Moreover, our study further explores the distinct predictive capabilities of different TyG-related indices. We found that TyG-WC and TyG-WHtR were more strongly associated with kidney stone risk compared to TyG-BMI, reinforcing the hypothesis that abdominal obesity, as measured by WC and WHtR, may better capture the metabolic disturbances underlying IR (40, 41). Recent evidence indicates that gastrointestinal hormones, particularly ghrelin secreted by the gastric fundus, play a pivotal role in glucose homeostasis and metabolic regulation (42). In obesity, the dysregulation of these hormones may contribute to insulin resistance and impaired glucose metabolism, both key components of metabolic syndrome that are strongly linked to kidney stone formation. Visceral adiposity, particularly in the abdominal region, has been strongly linked to metabolic syndrome and is believed to play a critical role in IR. Visceral fat is metabolically active, secreting pro-inflammatory cytokines that exacerbate systemic inflammation and disrupt renal calcium handling, which can lead to nephrolithiasis (43–46). This is supported by earlier studies indicating that WC, rather than overall BMI, may be a more reliable predictor of metabolic disorders and related complications, including kidney stones (47, 48).

The AUC values for TyG-related indices (ranging from 0.5815 to 0.6158) in our study suggest modest predictive capability for kidney stone formation. These relatively low AUC values likely reflect the complex and multifactorial nature of kidney stone disease, which involves diverse pathophysiological mechanisms, environmental influences, and genetic predispositions. While TyG-related indices may serve as accessible markers of insulin resistance and its potential role in kidney stone risk, they capture only one facet of this intricate pathogenic process. Other critical factors, such as dietary habits, fluid intake, urinary pH, mineral metabolism, and genetic predisposition, also play significant roles in stone formation but are not accounted for by these indices. Consequently, these markers should be viewed as complementary tools rather than standalone predictors for kidney stone risk assessment. Future studies integrating TyG-related indices with other established risk factors may enhance the overall predictive accuracy for kidney stone formation.

In addition to confirming the association between TyG-related indices and kidney stone formation, our study also identified a novel interaction between alcohol consumption and TyG-WHtR. This significant interaction suggests that lifestyle factors may modify the relationship between metabolic dysfunction and stone formation. This finding has not been well explored in previous studies but may indicate that alcohol consumption interacts with IR by exacerbating oxidative stress and altering lipid metabolism, both of which could influence stone risk (49–51). Studies (52–55) on alcohol’s effect on metabolic health suggest that while moderate alcohol consumption may have protective cardiovascular effects, excessive intake can lead to increased triglyceride levels and systemic inflammation, both of which could contribute to kidney stone formation. However, the precise mechanisms behind this interaction remain unclear, and further research is necessary to elucidate how alcohol modulates the risk of nephrolithiasis in individuals with high TyG-related indices.

Despite the valuable findings, our study has several limitations. The cross-sectional design prevents us from establishing a causal relationship between IR and kidney stone formation. Additionally, the use of self-reported kidney stone history introduces the potential for recall bias, which may affect the reliability of our results. Furthermore, the study population was limited to a single ethnic group, potentially impacting the generalizability of the findings to other populations. Moreover, several potential confounding factors were not evaluated in this study, including elevated serum uric acid levels, calcium metabolism disorders, diuretic use, and genetic predisposition. Recent studies (56–58) have demonstrated associations between these factors and kidney stone formation; therefore, their omission may have introduced bias into our findings. Future research should consider these potential confounding variables, employ longitudinal designs to establish causality, use objective measures of kidney stone incidence, and more comprehensively account for dietary and lifestyle factors. Expanding studies to include diverse populations will also enhance the applicability of the findings.

## 5 Conclusions

This study demonstrates a significant association between TyG-related indices (TyG-BMI, TyG-WC, TyG-WHtR) and kidney stone formation. These indices effectively reflect the link between IR and kidney stone risk. Future research should include cohort studies that account for additional lifestyle and genetic factors to provide a deeper understanding of these associations.

## Data Availability

The original contributions presented in the study are included in the article/supplementary material. Further inquiries can be directed to the corresponding author.
